# Influence of posterior tibial slope on sagittal knee alignment with comparing contralateral knees of anterior cruciate ligament injured patients to healthy knees

**DOI:** 10.1038/s41598-022-18442-y

**Published:** 2022-08-18

**Authors:** Yoshiaki Hiranaka, Hirotsugu Muratsu, Masanori Tsubosaka, Tomoyuki Matsumoto, Akihiro Maruo, Hidetoshi Miya, Ryosuke Kuroda, Takehiko Matsushita

**Affiliations:** 1 Department of Orthopaedic Surgery, Hyogo Prefectural Harima-Himeji General Medical Center, 3-264, Kamiya-cho, Himeji, 670-8560 Japan; 2 Department of Orthopaedic Surgery, Kobe University Graduate School of Medicine, 7-5-2, Kusunoki-cho, Chuou-ku, Kobe, 650-0017 Japan

**Keywords:** Medical research, Signs and symptoms

## Abstract

Posterior tibial slope (PTS) has been known to contribute to anterior–posterior knee stability and play an essential biomechanical role in knee kinematics. This study aimed to investigate the effect of PTS on single-leg standing sagittal knee alignment of the intact knee. This study included 100 patients with unilateral ACL injury knee (ACL injury group, 53 patients) or with the normal knee (control group, 47 patients). The single-leg standing sagittal alignment of the unaffected knees of the ACL injury group and normal knees of the control group were assessed radiographically with the following parameters: knee extension angle (EXT), PTS, PTS to the horizontal line (PTS-H), femoral shaft anterior tilt to the vertical axis (FAT), and tibial shaft anterior tilt to the vertical axis (TAT). PTS was negatively correlated with EXT and positively correlated with TAT. EXT was significantly larger in the ACL injury group, whereas TAT was smaller in the ACL injury group. Patients with larger PTS tend to stand with a higher knee flexion angle by tilting the tibia anteriorly, possibly reducing tibial shear force. Patients with ACL injury tend to stand with larger EXT, i.e., there is less preventive alignment to minimize the tibial shear force.

## Introduction

Anterior–posterior knee stability under weight-bearing conditions is dependent on several factors, including the anterior and posterior cruciate ligaments, menisci, and joint capsule^1–3^. Similarly, the articular surfaces of the tibiofemoral joint play an important role in controlling the biomechanical behavior of the joint along with the primary ligaments. In particular, the proximal tibial articular surface holds the distal femoral articular surface and functions as a load-bearing horizontal plane for balance and bipedal walking under weight-bearing conditions^4^. Posterior tibial slope (PTS), one of the morphological indicators of the proximal tibial articular surface, has been known to contribute to anterior–posterior knee stability and play an important biomechanical role in knee kinematics. In recent years, it has been reported that PTS affects the postoperative performance of knee joint surgeries such as anterior cruciate ligament (ACL) reconstruction^5–7^, high tibial osteotomy (HTO)^8,9^, and knee arthroplasty surgery, including knee arthroplasty surgery total and unicompartmental knee arthroplasties (UKA)^10,11^. Furthermore, anterior tibial translation and tibial shear force are known to increase as PTS increases during weight-bearing conditions, resulting in a higher risk of ACL injury^9,12,13^.

Several studies have reported on how the morphology of the proximal tibial articular surface affects knee alignment under weight-bearing conditions^14,15^. For example, in the coronal plane, the inclination of the articular surface in the medial compartment of the proximal tibia becomes more horizontal to the ground under weight-bearing conditions^14^. However, the influence of PTS on the sagittal alignment of the intact knees under weight-bearing conditions has rarely been studied. Therefore, we hypothesized that the sagittal knee alignment changes depending on PTS under weight-bearing conditions to control tibial share force caused by PTS in the intact knee. Thus, this study aimed to examine the effect of PTS on sagittal knee alignment in intact knees using plain lateral-view single-leg standing radiographs. In addition, we hypothesized that the knee alignment of patients with an ACL injury would be more strongly determined by their PTS. Therefore, we examined the characteristics of the sagittal knee alignment in patients with ACL injuries by comparing the difference between the unaffected side of the ACL-injured knees and normal knees.

## Materials and methods

### Patients

The current study was approved by the ethical committee of Steel Memorial Hirohata Hospital (IRB No. H30-135). Informed consent was obtained from all participants. All methods were performed in accordance with the relevant guidelines and regulations (Declaration of Helsinki). Patients with a unilateral ACL injury treated at our hospital between 2012 and 2018 were eligible for inclusion in this retrospective, case–control study. The inclusion criteria of ACL injury for this study were unilateral ACL injury and age of 16 to 40 years at the time of injury. All patients with ACL injuries had non-contact injuries. We excluded patients with diseases that cause joint inflammation, such as rheumatoid arthritis, with a history of trauma or surgery on the unaffected lower limb, with osteoarthritic (OA) change in either knee or knee flexion contracture on the unaffected side. ACL injuries were diagnosed by manual examination, including the Lachman test, pivot shift test, and anterior drawer test. All ACL injured patients underwent magnetic resonance imaging to confirm ACL tear.

As a control group, the contralateral knee of a patient with a unilateral meniscus injury was used as the normal knee. Patients with discoid meniscal injuries were excluded from this study because of the possibility of morphological tibial abnormalities^16^.

### Radiographic evaluation

**Figure 1:**
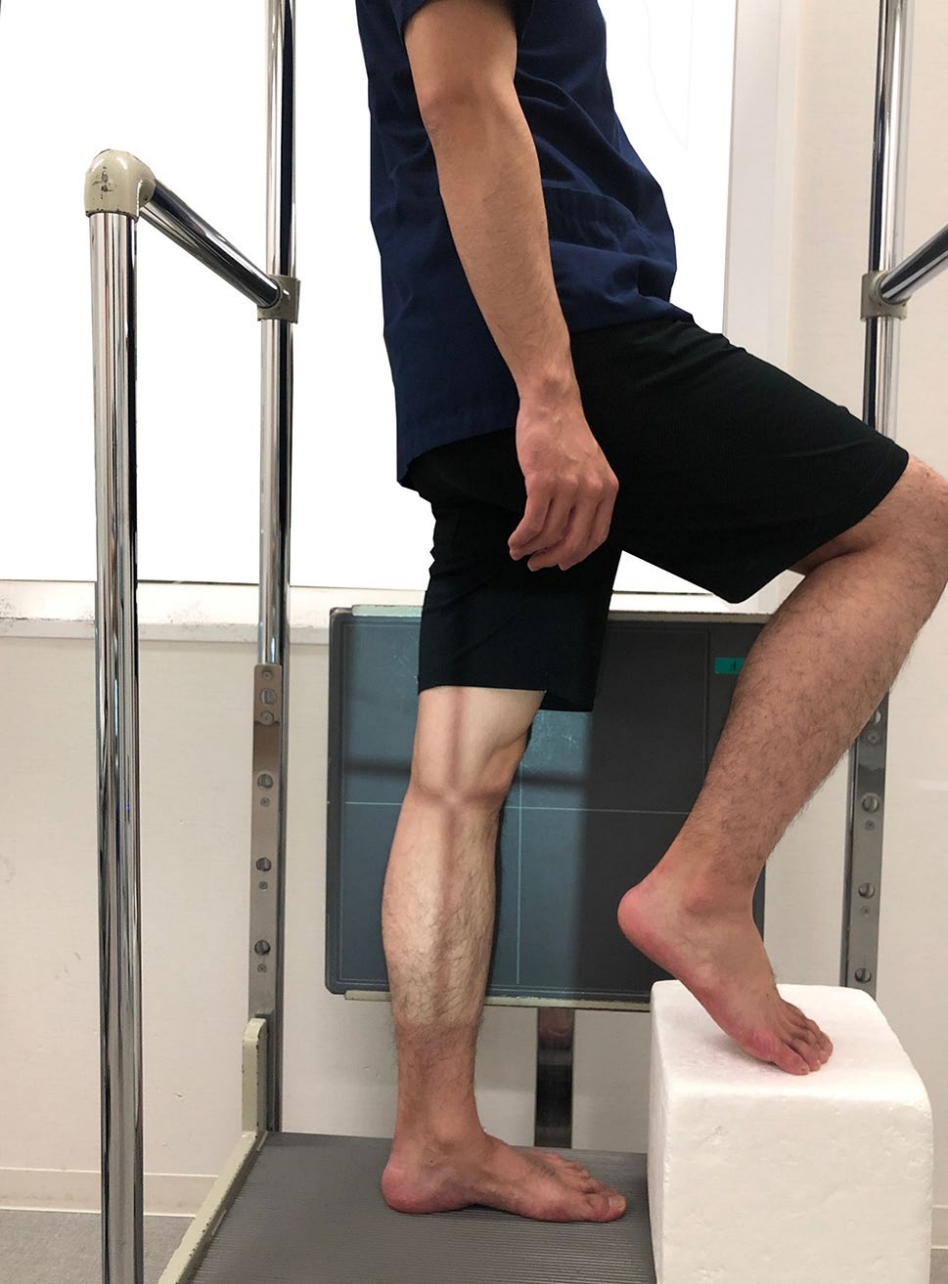
Procedure for taking the single-leg standing knee radiograph. A lateral-view single-leg standing knee radiograph was taken with the contralateral side of the foot placed on the footstool without weight-bearing.

A plain lateral-view single-leg standing radiograph of the knees was taken with the contralateral side of the foot placed on the footstool without weight-bearing. We instructed the radiologist to correct the patient to a natural single-leg standing position if the patient was weight-bearing on the footstool and the trunk was tilted forward. The radiograph cassette was set parallel to the ground, and the radiograph appropriately incident on the posterior condyle of the femur and femorotibial joint surface was used. The method of taking single-leg knee radiographs is shown in Fig. 1. The radiographic examination was performed for bilateral knees as a routine radiographic examination for patients who visited our hospital with a complaint of a knee symptom.

**Figure 2:**
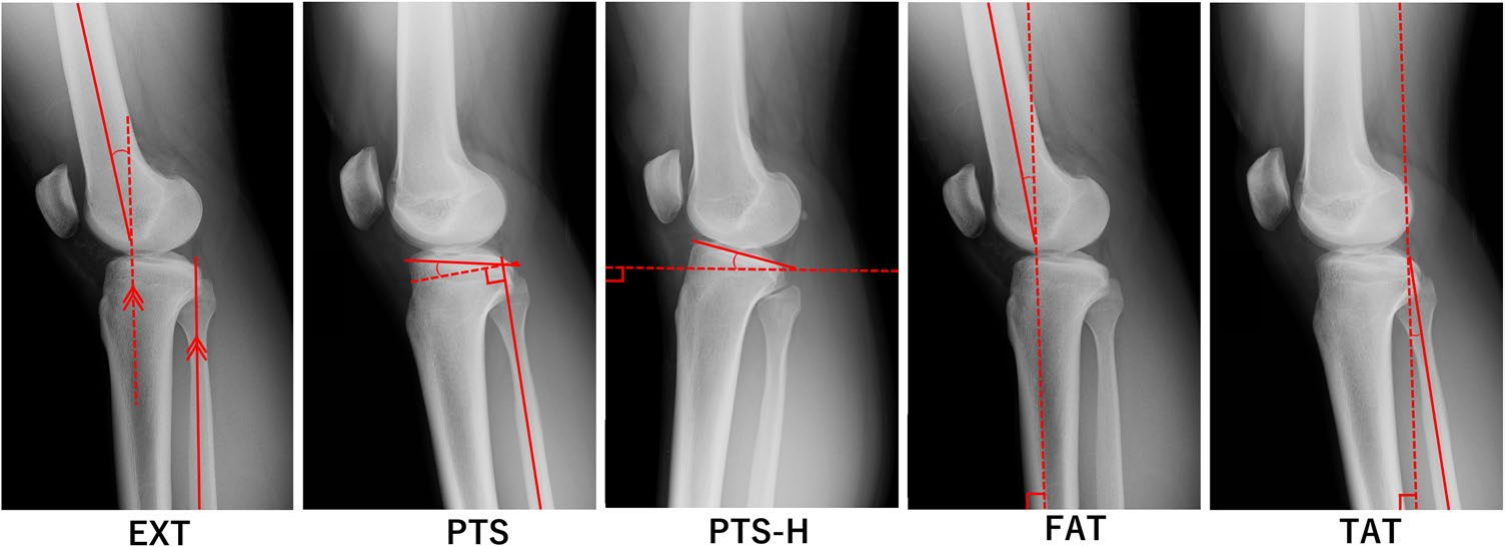
Measurement of the five radiographic parameters: EXT, PTS, PTS-H, FAT, and TAT. EXT, knee extension angle, with the hyperextended position denoted as a positive value and the flexion position as a negative value; PTS, posterior tibial slope; PTS-H, posterior tibial slope to the horizontal line; FAT, femoral shaft anterior tilt to the vertical axis, with the forward tilt to the perpendicular line as the positive value; TAT, tibial shaft anterior tilt to the vertical axis, with the backward tilt to the perpendicular line as the negative value.

In this study, the radiographic measurements were performed using radiographs of the unaffected knees of the ACL injury group and control groups’ unaffected knees. We evaluated the following radiographic parameters: knee extension angle (EXT), PTS, PTS to the horizontal line (PTS-H), femoral shaft axis anterior tilt to the vertical axis (FAT), and tibial shaft axis anterior tilt angle to the vertical axis (TAT). Detailed measurement methods are shown in Fig. 2. The mechanical tibial shaft axis for EXT, PTS, and TAT was measured by substituting the proximal fibular shaft axis^17^. For EXT, the hyperextension position was denoted with a positive value and the flexion position with a negative value. FAT and TAT were defined as positive for a forward tilt to the perpendicular line and negative for a backward tilt to the perpendicular line. Measurements of these radiographic parameters were performed using the working software of a picture archiving and communication system.

### Statistical analysis

All data are shown as mean ± standard deviation. The comparison of the number of male and female patients between the ACL injury group and the control group was performed using a chi-square test, and the comparison of age, height, body weight, body mass index (BMI), and radiographic parameters between the ACL injury group and the control group was performed using an unpaired t-test. The effects of the PTS on the other four parameters were analyzed with a simple linear regression analysis of the ACL injury group, the control group, and all the subjects (ACL injury group plus control group). Statistical analysis was performed using SPSS for Windows version 20.0 (IBM Corp., Armonk, NY, USA). A *P* value < 0.05 was considered to indicate a statistically significant difference. To assess the reliability of the measurements of radiographic parameters, two observers measured 30 knees randomly selected from the 100 knees; each measurement was mainly performed twice in a blinded manner by an independent orthopaedic surgeon (HM) and performed once by another orthopaedic surgeon (MT). The degree of measurement reliabilities was assessed using the intraclass correlation coefficient (ICC). The ICCs for intra-rater and inter-rater agreement were 0.974 (range 0.958–0.987) and 0.965 (range 0.922–0.983) for all radiographic parameters, respectively. A statistical priori power analysis was performed to determine the sample size based on the difference in EXT between the two groups. In this analysis, G*Power software (version 3.1.9.4; Heinrich Heine Universität Düsseldorf, DE) was used with a prespecified significance level of α < 0.05, a power level of 95%, and an effect size based on the results of the pilot study with 15 cases (effect size d = 0.72). The estimated sample size was 43 patients.

## Results

A total of 100 knees with unaffected side knees of unilateral ACL injured patients (ACL injury group, 53 cases) and normal knees (control group, 47 cases) were evaluated. The participants consisted of 56 males and 44 females with a mean age of 23.6 ± 0.8 years (range 13–40 years).

The patient demographics, including sex, age, height, body weight, and BMI, are shown in Table 1. Sex, age, and height were not significantly different between the ACL injury and control groups, whereas body weight and BMI were significantly greater in the ACL injury group.

**Table 1:** Patient demographics.

	All the subjects (n = 100)	ACL injury group (n = 53)	Control group (n = 47)	*P* value
Sex (male/female)	56/44	31/22	25/22	0.594
Age (years)	23.6 ± 7.5	22.8 ± 6.8	24.5 ± 8.2	0.273
Height (cm)	166.7 ± 8.8	166.1 ± 7.9	167.4 ± 9.7	0.467
Weight (kg)	64.4 ± 12.4	66.7 ± 11.9	61.8 ± 12.5	0.048*
BMI (kg/m^2^)	23.1 ± 3.6	24.1 ± 3.7	21.9 ± 3.0	0.002*

Data are shown as the mean ± standard deviation. ACL, anterior cruciate ligament; BMI, body mass index. *P < 0.05, statistically significant.

Each radiographic parameter in all the subjects was EXT: 2.4 ± 6.2°, PTS: 9.5 ± 3.0°, PTS-H: 8.1 ± 3.9°, FAT: 3.8 ± 4.7° and TAT: 1.4 ± 4.2°. Table S1 shows the details of radiographic parameters for all patients.

**Figure 3:**
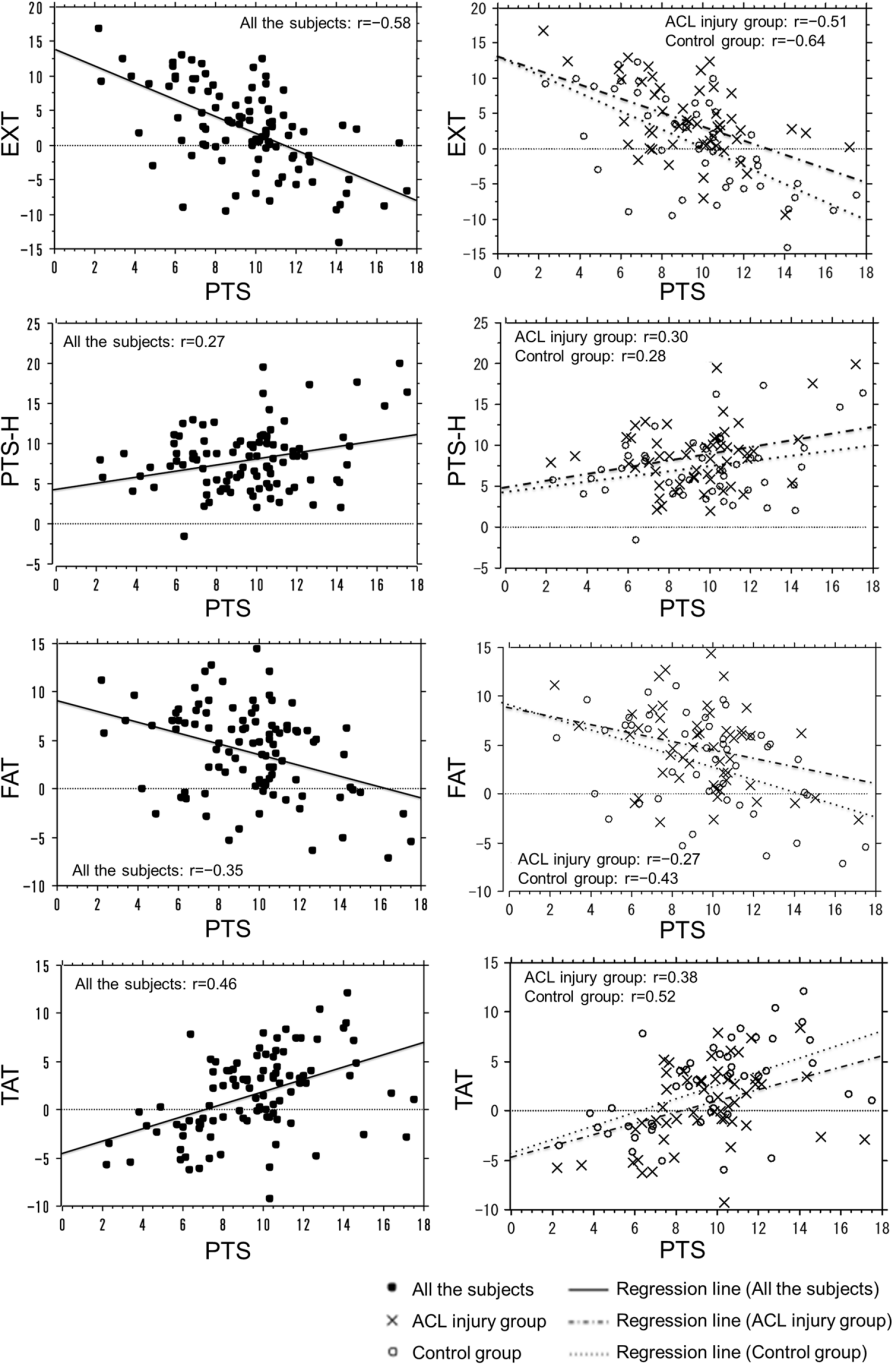
Regression lines for the relationship between the PTS and the other four parameters (EXT, PTS-H, FAT, and TAT). EXT, knee extension angle; PTS, posterior tibial slope; PTS-H, posterior tibial slope to the horizontal line; FAT, femoral shaft anterior tilt to the vertical axis; TAT, tibial shaft anterior tilt to the vertical axis; ACL, anterior cruciate ligament.

**Figure 4:**
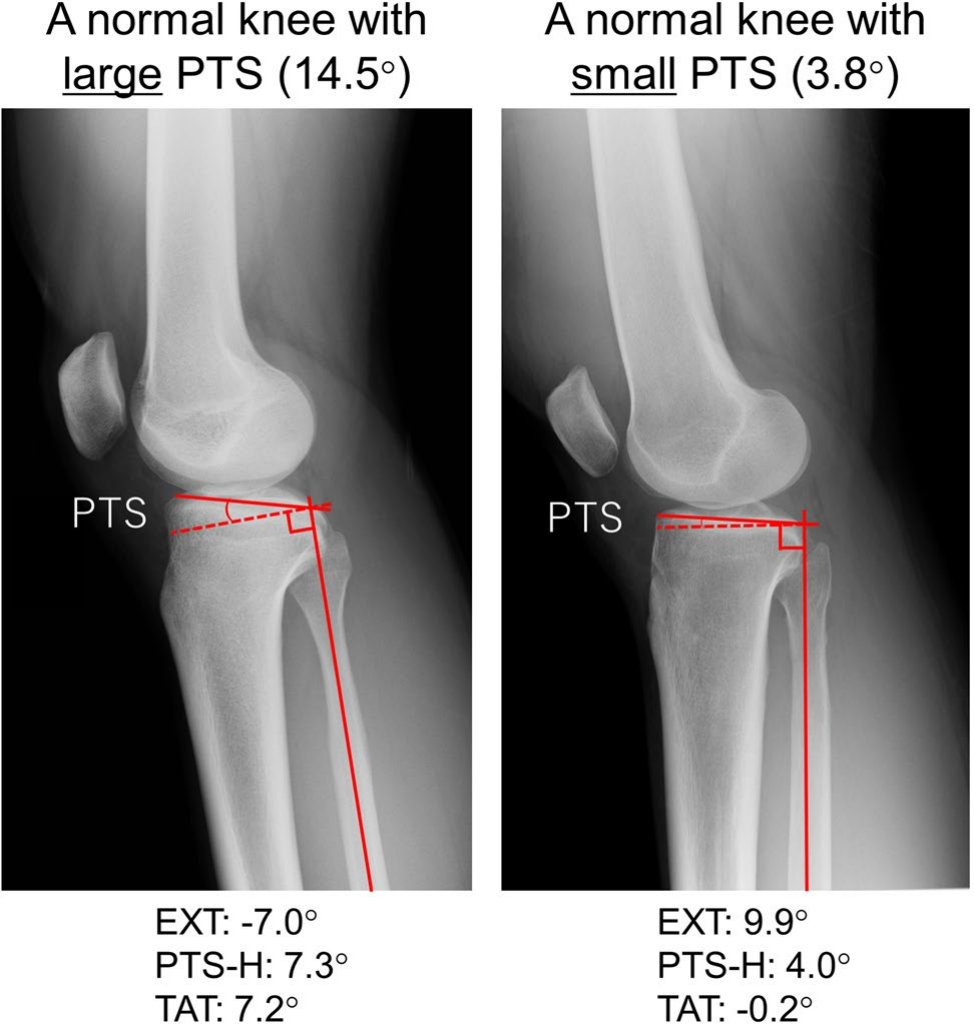
Typical lateral-view radiographs of a normal knee with a large PTS, and with a small PTS. The unaffected side of the knees is shown. The patient with a large PTS (left radiograph) was standing with the knee slightly flexed and the proximal tibial surface level to the ground. PTS, posterior tibial slope; PTS-H, posterior tibial slope to the horizontal line; TAT, tibial shaft anterior tilt to the vertical axis; ACL, anterior cruciate ligament.

The correlations between PTS and the other four parameters are shown in Table 2 and Fig. 3. PTS had a significant moderate negative correlation with EXT in all the subjects, in the ACL injury group and the control group (all the subjects: r = − 0.58, *P* < 0.001, ACL injury group: r = − 0.51, *P* < 0.001, control group: r = − 0.64, *P* < 0.001). PTS also had a significant moderate positive correlation with TAT in all the subjects, the ACL injury group, and the control group (all the subjects: r = 0.46, *P* < 0.001, ACL injury group: r = 0.38, *P* = 0.005, control group: r = 0.52, *P* < 0.001). There was no significant correlation between PTS and PTS-H in the control group; however, there were significantly low correlations in all the subjects and ACL injury group (all the subjects: r = 0.27, *P* = 0.007, ACL injury group: r = 0.30*, P* = 0.031). There was no significant correlation between PTS and FAT in the ACL injury group; however, there were significant low-to-moderate correlations in all the subjects and the control groups (all the subjects: r = − 0.35, *P* < 0.001, control group: r = − 0.43, *P* = 0.003). PTS-H tended to increase in patients with large PTS. Figure 4 shows the typical lateral-view radiographs of a normal knee with a large PTS versus a knee with a small PTS.

**Table 2:** Results of the simple linear regression analysis of the relationship between PTS and the other four parameters (EXT, PTS-H, FAT, and TAT).

	All the subjects		ACL injury group		Control group	
	r	*P* value	r	*P* value	r	*P* value
EXT (°)	− 0.58	< 0.001*	− 0.51	< 0.001*	− 0.64	< 0.001*
PTS-H (°)	0.27	0.007*	0.30	0.031*	0.28	0.062
FAT (°)	− 0.35	< 0.001*	− 0.27	0.054	− 0.43	0.003*
TAT (°)	0.46	< 0.001*	0.38	0.005*	0.52	< 0.001*

ACL, anterior cruciate ligament; EXT, knee extension angle; PTS, posterior tibial slope; PTS-H, posterior tibial slope to the horizontal line; FAT, femoral shaft axis anterior tilt to the vertical axis; TAT, tibial shaft axis anterior tilt angle to the vertical axis; r, correlation coefficient. *P < 0.05, statistically significant.

**Figure 5:**
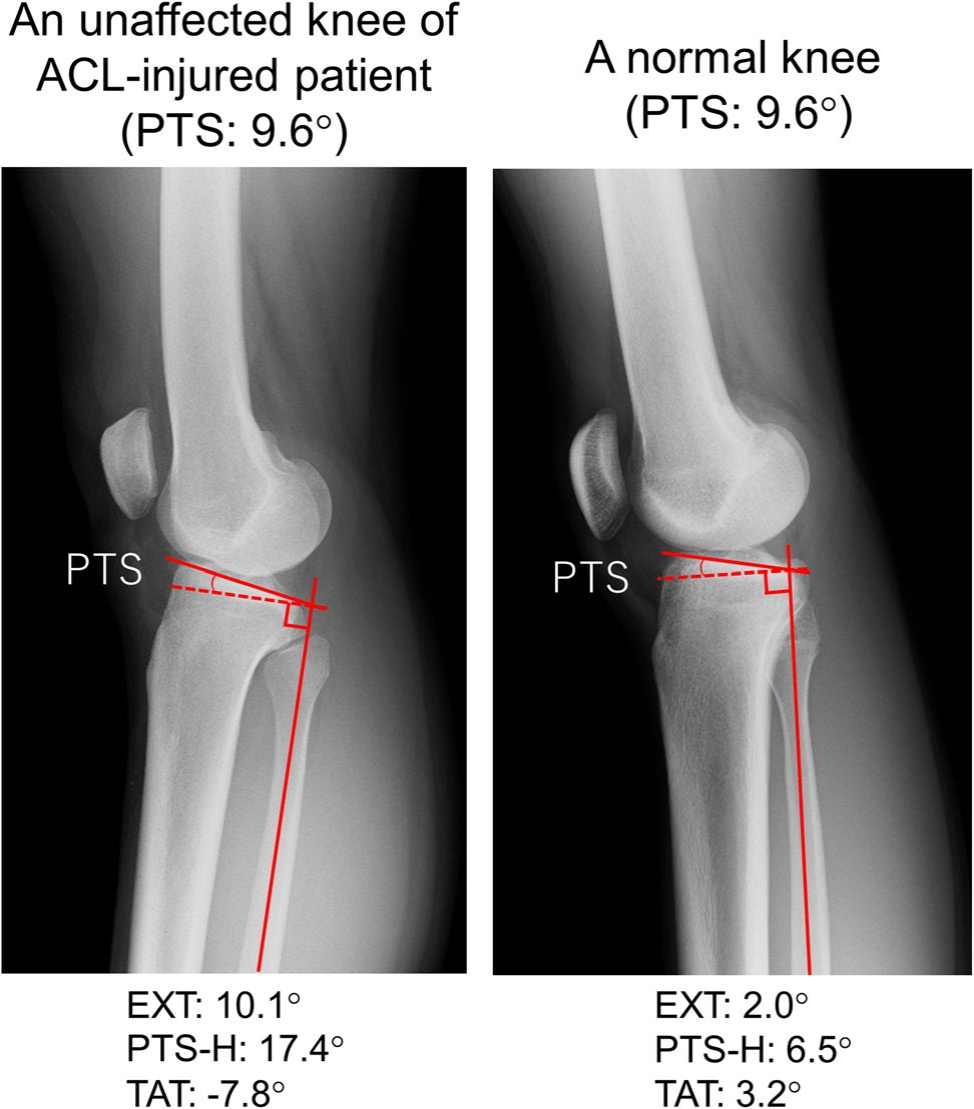
Typical lateral-view radiographs of an unaffected knee of an ACL-injured patient (left) and a normal knee (right). The unaffected side of the knees is shown. The patient with the ACL injury was standing with the knee hyperextended and with a large posterior slope to the ground, resulting in less anterior knee tilt compared with that of the patient with a normal knee. PTS, posterior tibial slope; PTS-H, posterior tibial slope to the horizontal line; TAT, tibial shaft anterior tilt to the vertical axis; ACL, anterior cruciate ligament.

The comparison among radiographic parameters is presented in Table 3. The mean PTS was not significantly different between the ACL injury group and the control group (ACL injury group: 9.4 ± 2.7° vs. control group: 9.6 ± 3.3°, *P* = 0.651). Meanwhile, EXT was significantly larger in the ACL injury group than in the control group (3.9 ± 5.3° vs. 0.7 ± 6.8°, *P* = 0.001), and TAT was significantly smaller in the ACL injury group than in the control group (0.6 ± 4.0° vs. 2.3 ± 4.4°, *P* = 0.045). Although not significant, the mean PTS-H was greater in the ACL injury group than in the control group (8.7 ± 3.9° vs. 7.3 ± 3.9°, *P* = 0.069). Figure 5 shows the typical lateral-view radiographs of an unaffected knee of an ACL-injured patient and a normal knee.

**Table 3:** Comparisons of the radiographic parameters among all the subjects, the ACL injury group, and the control group.

	All the subjects	ACL injury group	Control group	*P* value
EXT (°)	2.4 ± 6.2	3.9 ± 5.3	0.7 ± 6.8	0.001*
PTS (°)	9.5 ± 3.0	9.4 ± 2.7	9.6 ± 3.3	0.651
PTS-H (°)	8.1 ± 3.9	8.7 ± 3.9	7.3 ± 3.9	0.069
FAT (°)	3.8 ± 4.7	4.5 ± 4.5	3.1 ± 4.9	0.132
TAT (°)	1.4 ± 4.2	0.6 ± 4.0	2.3 ± 4.4	0.045*

Data are shown as the mean ± standard deviation. ACL, anterior cruciate ligament; EXT, knee extension angle; PTS, posterior tibial slope; PTS-H, posterior tibial slope to the horizontal line; FAT, femoral shaft axis anterior tilt to the vertical axis; TAT, tibial shaft axis anterior tilt angle to the vertical axis. *P < 0.05, statistically significant.

## Discussion

One of the most pertinent findings of this study was that as PTS increased, EXT decreased, and TAT increased during single-leg standing. In other words, patients with increased PTS tended to stand with increased knee flexion and forward tilting of the tibia. These results support our hypothesis that the sagittal knee alignment is associated with PTS under weight-bearing conditions. It is assumed that PTS and sagittal alignment have a mutual influence on each other.

We used a single-leg standing radiograph for evaluation for the three following reasons: First, evaluation using single-leg standing radiographs for bilateral knees has been routinely performed for knee-related diseases. Therefore, we did not have to take any additional radiographs for this study. Second, single-leg radiographs are more likely to reduce the contralateral knee's influence than the standard weight-bearing standing radiographs. This is because standard weight-bearing standing radiographs cannot eliminate the effect of the weight-bearing of the contralateral leg, and the amount of loading is expected to change the alignment of the knee. Further, we also aimed to investigate the postural characteristics of ACL-injured patients while they stood on the side of the healthy knee. The weight-bearing conditions were expected to differ between single-leg and standard weight-bearing standing radiographs. We believed that the mechanical conditions of the knee would be more consistent with single-leg standing than the standard two-leg standing position. Therefore, we evaluated the single-leg radiographs’ alignment, which is considered the most common posture for patients to adopt when injured.

As for the effect of PTS under weight-bearing conditions, tibial shear force, anterior tibial translation, and ACL force increased as PTS increased during standing and walking^9,12,13^. To control tibial shear force, patients reduced their PTS perpendicularly against gravity by leveling the proximal articular surface of the tibia to the ground by tilting the tibial shaft more anteriorly and bending the knee. This result of our study supported the hypothesis that the knee flexes according to PTS to reduce tibial shear force under weight-bearing conditions. This mechanism has not been described before and will be important in cruciate-retaining surgeries that may change PTS such as UKA and HTO. For example, in UKA, a very large PTS can lead to continuous slight knee flexion under weight-bearing, resulting in knee flexion contractures and shortening of the hamstring muscles.

Another important result of this study was that EXT was significantly larger while TAT was smaller in unaffected knees of ACL injured patients than in normal knees. As for the relationship between PTS and ACL injury, a meta-analysis reported that five of the six studies on radiographs of PTS showed significant differences between controls and those with ACL injury^13^. Meanwhile, in our study, PTS was not significantly different between the ACL injury and control groups. Nevertheless, it is interesting to note that the ACL injury group demonstrated smaller TAT and larger EXT despite no significant differences in PTS between the two groups. In other words, the ACL injury group was loaded with a smaller TAT and more knee extension than the control group, and this demonstrated the characteristic sagittal knee alignment of patients with an ACL injury, which is thought to be mechanically disadvantageous in reducing the influence of the tibial forward vector on the vertical load caused by the posterior slope of the tibial plateau. This disadvantageous alignment indicated that ACL-injured patients stood with a less preventive mechanism, even on the side of the healthy knee, to flex the knee depending on PTS and obtain anterior–posterior knee stability compared with that of the control group. Although this alignment may be influenced by joint laxity, which is commonly observed in patients with ACL injuries, this standing position may be an indicator of patients at risk for ACL injury. While not statistically significant, the PTS-H tended to be larger in the ACL injury group. This result supports the second hypothesis that the knee alignment of ACL-injured patients may be more strongly determined by their PTS; in other words, ACL-injured patients had a poor mechanism of parallelizing the tibial plateau to the ground to obtain the anterior knee stability.

Owusu-Akyaw et al.^18^ reported that landing on an extended knee is a high risk for ACL injury regardless of sex. Knee extension position is accompanied by an increased anterior tibial translation, internal tibial rotation, and valgus rotation in the predicted place of ACL injury. Joint laxity is one of the risk factors for ACL injury^19^, and the laxity of a knee joint in ACL-injured patients may have contributed to the tendency for knee extension during standing. However, we did not measure the exact maximum knee extension angle in these patients. However, the fact was that ACL-injured patients had a lower ability to compensate for their laxity during standing. We believe that the analysis of sagittal knee alignment, based on parameters such as PTS, EXT, and TAT, using simple single-leg standing radiographs may be helpful in the study of risk factors for ACL injury.

Our study has several limitations. The contralateral knee of patients with a unilateral meniscus injury was defined as the normal knees, but they might differ from completely normal knees. Sagittal knee alignment was measured with radiographs, which are frequently used in the clinical situation; however, no detailed investigation has been performed on the reproducibility of this measurement method. We evaluated only the alignment of the distal femur and proximal tibia or fibula for the sagittal knee alignment and did not evaluate long-leg radiographs. The main reason for this was that we needed to take long leg lateral-view radiographs with the hip joint arbitrarily rotated externally (similar to the standing Lowenstein position). The femoral head did not overlap with the pelvis. Therefore, it would be a non-physiological single-leg standing position. This study took standard lateral knee radiographs instead of long-leg radiographs to evaluate the knee alignment while reproducing the physiological single-leg standing position. In addition, the installation angle of the cassette was adjusted using a spirit level so that the cassette was installed horizontally to the ground. Short lateral knee radiographs have commonly been used clinically and in many studies to measure knee alignment. For this study, subjects who could efficiently perform single-leg standing with their healthy-side knee were subjected to lateral knee radiography under mechanical conditions close to walking. For these reasons, this analysis focused on comparing the sagittal knee alignment under physiological conditions between the two relative groups. We did not intend to evaluate knee alignment using the lower limb load axis with an arbitrarily controlled position. Instead of long-leg radiographs, short lateral knee radiographs with a cassette size of 17 × 14 inches had enough filming coverage in this study on the Japanese population. However, we could not include the ankle joint in our assessment because of the short-film evaluation. TAT may be affected by the alignment or range of motion of a tibiotalar or subtalar joint. Finally, we did not evaluate the difference in tightness and strength of the hamstrings or quadriceps, and this difference may have affected the lateral alignment of the standing knee.

## Conclusions

Patients with a larger PTS tend to stand with a higher knee flexion angle by tilting the tibia anteriorly, possibly reducing tibial shear force. Patients with ACL injury tend to stand with larger EXT; there is less preventive alignment to reduce tibial shear force.

## Supplementary Information

Supplementary Table S1.

## Data Availability

The datasets analyzed during the current study are available from the corresponding author upon request.
